# Pre‐diagnostic immunological markers of bacterial translocation and liver cancer risk: A nested case–control analysis of 12 prospective cohorts

**DOI:** 10.1002/ijc.70201

**Published:** 2025-10-23

**Authors:** Cody Z. Watling, Peter T. Campbell, Barry I. Graubard, Yanyu Wang, Andrew T. Gewirtz, Xuehong Zhang, Matthew J. Barnett, Julie E. Buring, Yu Chen, A. Heather Eliassen, J. Michael Gaziano, Jonathan N. Hofmann, Wen‐Yi Huang, Jae H. Kang, Jill Koshiol, Erikka Loftfield, I‐Min Lee, Steven C. Moore, Lorelei A. Mucci, Marian L. Neuhouser, Christina C. Newton, Mark P. Purdue, Howard D. Sesso, Martha Shrubsole, Rashmi Sinha, Lesley Tinker, Matthew Triplette, Caroline Y. Um, Kala Visvanathan, Eleanor L. Watts, Jean Wactawski‐Wende, Walter Willett, Fen Wu, Wei Zheng, Dinesh Barupal, Jessica L. Petrick, Katherine A. McGlynn

**Affiliations:** ^1^ Division of Cancer Epidemiology and Genetics National Cancer Institute Rockville Maryland USA; ^2^ Department of Epidemiology & Population Health Albert Einstein College of Medicine New York New York USA; ^3^ Applied Developmental Research Directorate, Leidos Biomedical Research Inc Frederick National Laboratory for Cancer Research, National Cancer Institute Frederick Maryland USA; ^4^ Center for Inflammation, Immunity and Infection Institute for Biomedical Sciences, Georgia State University Atlanta Georgia USA; ^5^ School of Nursing Yale University New Haven Connecticut USA; ^6^ Cancer Prevention Program, Division of Public Health Sciences Fred Hutchinson Cancer Center Seattle Washington USA; ^7^ Division of Preventive Medicine Brigham and Women's Hospital, Harvard Medical School Boston Massachusetts USA; ^8^ Department of Epidemiology Harvard T.H. Chan School of Public Health Boston Massachusetts USA; ^9^ Department of Population Health NYU Grossman School of Medicine New York New York USA; ^10^ Channing Division of Network Medicine, Department of Medicine Brigham and Women's Hospital and Harvard Medical School Boston Massachusetts USA; ^11^ Department of Population Science American Cancer Society New York New York USA; ^12^ Division of Epidemiology Vanderbilt Epidemiology Center, Vanderbilt‐Ingram Cancer Center Nashville Tennessee USA; ^13^ Johns Hopkins School of Medicine and Bloomberg School of Public Health Baltimore Maryland USA; ^14^ Department of Epidemiology and Environmental Health University at Buffalo Buffalo New York USA; ^15^ Department of Environmental Medicine Icahn School of Medicine at Mount Sinai New York New York USA; ^16^ Slone Epidemiology Center at Boston University Boston Massachusetts USA

**Keywords:** endotoxin, flagellin, gut dysbiosis, hepatocellular carcinoma, intrahepatic cholangiocarcinoma

## Abstract

The gut‐liver axis may play an important role in hepatocarcinogenesis. However, limited prospective research has explored associations with liver cancer risk. We conducted a nested case–control study based in 12 prospective cohort studies from across the United States, which included 867 cases of liver cancer and 867 matched controls. We measured bacterial translocation markers, specifically immunoglobulin (Ig) A, IgG, and IgM against lipopolysaccharide and flagellin; soluble CD14 (a co‐receptor for lipopolysaccharide); and lipopolysaccharide‐binding protein. Multivariable conditional logistic regression was used to estimate adjusted odds ratios (OR) and 95% confidence intervals (CI) between bacterial translocation marker concentrations per doubling in concentrations and liver cancer risk.

Lipopolysaccharide‐binding protein concentrations were most strongly associated with higher liver cancer risk (OR per doubling in concentrations: 1.48, 95% CI: 1.23–1.79). Concentrations of anti‐flagellin IgA (1.13, 1.01–1.28) and IgG (1.13, 1.01–1.28), anti‐lipopolysaccharide IgG (1.20, 1.01–1.42), and soluble CD14 (1.12, 1.01–1.24) were also associated with liver cancer risk. When analyses were separated into hepatocellular carcinoma (HCC, *N* = 436 cases) and intrahepatic cholangiocarcinoma (ICC, *N* = 110 cases), no evidence of heterogeneity was observed except for lipopolysaccharide‐binding protein concentrations, which were positively associated with HCC (1.77, 1.34–2.33) but not ICC (0.67, 0.37–1.22; *p*‐heterogeneity = .003). Associations did not differ by time to liver cancer diagnosis or other subgroups. These findings support the role of gut barrier dysfunction in hepatocarcinogenesis, necessitating further research to understand the complex interplay among the mechanisms and risk factors disrupting the gut barrier, microbiota, and liver cancer.

## Graphical abstract

What's new?

Disruption of the gut–liver axis has been suggested to play an important role in liver carcinogenesis. However, limited prospective research has explored associations between potentially harmful gut‐derived bacterial products and liver cancer risk. In this nested case–control study, pre‐diagnostic immunological markers of bacterial translocation, measured an average of 12 years before diagnosis, were positively associated with liver cancer risk. Lipopolysaccharide‐binding protein concentrations were associated with hepatocellular carcinoma, but not intrahepatic cholangiocarcinoma. These findings point to modulation of the gut barrier as a potential strategy for reducing liver cancer risk.

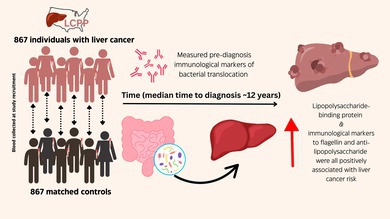

**Abbreviations**
BMI: body mass index
BWHS: Black Women's Health Study
CARET: Beta‐Carotene and Retinol Efficacy Trial (CARET)
CI: confidence intervals
CLUE: Campaign against Cancer and Stroke Study I and II (CLUE)
CPS‐II: Cancer Prevention Study‐II Nutrition Cohort (CPS‐II)
FDR: false discovery rate
HbsAg: hepatitis B surface antigen
HBV: hepatitis B virus
HCC: hepatocellular carcinoma
HCV: hepatitis C virus
HPFS: Health Professionals Follow‐Up Study
ICC: intrahepatic cholangiocarcinoma
ICD‐10: International Classification of Diseases 10th edition
Ig: Immunoglobulin
LBP: Lipopolysaccharide‐binding protein
LCPP: Liver Cancer Pooling Project
LPS: lipopolysaccharide
MAMP: microorganism‐associated molecular pattern
MASLD: Metabolic dysfunction–associated steatotic liver disease
NHS: Nurses' Health Study
NYUWHS: New York University Women's Health Study
OR: odds ratio
PHS: Physicians' Health Study
PLCO: Prostate, Lung, Colorectal and Ovarian Cancer Screening Trial
SCCS: Southern Community Cohort Study
sCD14: soluble CD14
TLR4: Toll‐like receptor 4
TLR5: Toll‐like receptor 5
WHI: Women's Health Initiative
WHS: Women's Health Study

## 1 INTRODUCTION

Liver cancer has been a leading cause of cancer mortality globally for the past two decades.^1^, ^2^ The two main histological subtypes are hepatocellular carcinoma (HCC), which accounts for 80%–85% of cases, and intrahepatic cholangiocarcinoma (ICC), which consists of 10%–15% of cases. Despite well‐established risk factors for liver cancer, including chronic infection with hepatitis B virus (HBV) or hepatitis C virus (HCV),^3^, ^4^ excessive alcohol consumption,^5^ smoking,^6^ and metabolic conditions such as obesity and diabetes^7^—all of which contribute to hepatic inflammation and can progress to liver disease and ultimately liver cancer—only 60% of cases are attributable to these risk factors,^8^ suggesting additional etiological risk factors remain to be elucidated.

One of the mechanisms that has been hypothesized to be an important modulator of liver cancer development is disruption of the gut‐liver axis.^9^, ^10^, ^11^ Aberrant gut microbiota have been implicated in chronic liver disease and cirrhosis,^9^, ^12^ and HCC.^13^ An important component of the gut‐liver axis is the gut barrier function. The gut barrier serves a dual function, permitting the selective permeability of essential nutrients from the intestinal lumen into systemic circulation via the hepatic portal vein, while restricting the translocation of potentially harmful factors, including bacteria and their associated endotoxins, into the blood. In animal models, gut dysbiosis has been shown to promote intestinal inflammation, which compromises epithelial barrier integrity and facilitates the translocation of microorganism‐associated molecular patterns (MAMPs), including lipopolysaccharide (LPS), an endotoxin derived from the outer membrane of gram‐negative bacteria, and flagellin, the structural component of bacterial flagella.^14^ Overabundance of bacterial LPS and flagellin and exposure to bacterial products have been shown to promote chronic inflammation and oxidative stress of the liver in animal models.^15^, ^16^ Lipopolysaccharide‐binding protein (LBP) and soluble CD14 (sCD14) may also be associated with liver cancer risk.^17^, ^18^ LBP, an acute‐phase protein primarily synthesized by hepatocytes in response to endotoxemia, binds to LPS to form an LPS–LBP complex.^19^ This complex is subsequently recognized by sCD14, which facilitates immune signaling and triggers an inflammatory response. Higher levels of LBP and sCD14 have been associated with liver injury and inflammation,^11^, ^20^ although associations with liver cancer remain unclear.^17^, ^18^

Despite the growing recognition of gut‐liver interactions in liver disease, epidemiologic studies evaluating the relationship between biomarkers of gut‐derived bacterial products and liver cancer risk remain limited.^17^, ^18^, ^21^ Two European cohorts found that antibodies to LPS and flagellin were positively associated with liver cancer risk,^18^, ^21^ whereas an analysis in a cohort from Taiwan found positive associations between LBP and anti‐flagellin in HBV‐associated HCC.^17^ The prior studies had fewer than 250 liver cancer cases, which may have limited the statistical power, and none examined ICC.

The objective of this study was to assess the associations between immunological markers of the bacterial translocation products, namely, anti‐flagellin, anti‐LPS, LBP, and sCD14, and liver cancer risk and subtypes of liver cancer in 12 prospective studies from the United States.

## 2 METHODS

### 2.1 Study population

This study utilized data from prospective cohorts in the Liver Cancer Pooling Project (LCPP). Details on the LCPP have been previously described.^22^ Briefly, the LCPP consists of North American‐based prospective cohort studies that are part of the National Cancer Institute Cohort Consortium. For this analysis, LCPP cohorts that collected pre‐diagnostic blood samples from participants were invited to participate in this collaborative analysis, resulting in the inclusion of 12 studies: the Black Women's Health Study (BWHS),^23^ Beta‐Carotene and Retinol Efficacy Trial (CARET),^24^ Campaign against Cancer and Stroke Study I & II (CLUE),^25^ Cancer Prevention Study‐II Nutrition Cohort (CPS‐II),^26^ Health Professionals Follow‐Up Study (HPFS),^27^ Nurses' Health Study (NHS),^28^ New York University Women's Health Study (NYUWHS),^29^ Physicians' Health Study (PHS),^30^ Prostate, Lung, Colorectal and Ovarian Cancer Screening Trial (PLCO),^31^ Southern Community Cohort Study (SCCS),^32^ Women's Health Initiative (WHI),^33^ and Women's Health Study (WHS).^34^ Table S1 provides further details on the recruitment periods and timing of blood sample collection for each cohort.

### 2.2 Case ascertainment

Diagnoses of incident liver cancer after blood draw were ascertained via linkages to state cancer registries or medical/pathology record review. Liver cancer was identified using the International Classification of Disease (ICD) 9th edition topography codes: 155.0–155.1 or 10th edition topography code: C22. If available, ICD‐O‐3 histology codes were used to determine liver cancer histological subtypes, specifically HCC: 8170–8175 and ICC: 8032–8033, 8041, 8050, 8070–8071, 8140–8141, 8160, 8260, 8480, 8481, 8490. In total, 436 cases of HCC and 110 cases of ICC were identified, with 321 cases being of unrecorded histology.

### 2.3 Control selection

In each cohort, the liver cancer cases were individually matched to controls at a 1:1 ratio using incident density matching within risk sets using follow‐up time as the underlying time metric in the same cohort (i.e., a control was a participant who could develop liver cancer in the future). Matching criteria included age at recruitment (same year), race/ethnicity, sex, and the date of baseline blood collection (within a fixed 3‐month calendar period).

## 3 LABORATORY ANALYSIS

Each cohort provided either serum or plasma samples that had been collected at recruitment. Details regarding the blood sample collection and storage across the cohorts are provided in Table S1. For quantifying bacterial translocation markers, samples were sent to the labs in matched pairs, with case/control status masked and each case/control pair within the same cohort run in the same analytical batch.

Antibodies to LPS and flagellin, specifically IgA, IgG, and IgM, were measured in the Gewirtz Lab at Georgia State University (Atlanta, Georgia) via a custom‐made ELISA, which has been previously described.^35^ Further details can be found in the Supporting Information.

LBP and sCD14 were measured in the Wang lab at the Frederick National Laboratory for Cancer Research (Frederick, Maryland). Further details can be found in the Supporting Information.

LBP was quantified using the R&D Systems DuoSet ELISA kit (Cat# DY870–05 and DY008), whereas sCD14 levels were measured using the R&D Systems Quantikine kit (Cat# CD140). Further details can be found in the Supporting Information.

Some participants (*n* = 553 [32% of participants], of which 286 were cases [33% of cases]) had information available on HCV and HBV infection status. Specifically, hepatitis B surface antigen (HBsAg) was previously assayed using the Bio‐Rad GS HBsAg 3.0 enzyme immunoassay (Bio‐Rad Laboratories, Redmond, WA, USA), and antibody to HCV (anti‐HCV) was assessed using the Ortho HCV Version 3.0 ELISA test system (Ortho‐Clinical Diagnostics, Inc., Raritan, NJ, USA).

### 3.1 Statistical analyses

Baseline characteristics of participants were summarized by case and control status across all the cohorts. All bacterial translocation markers were log‐transformed to approximate a normal distribution. If there were missing values for specific bacterial translocation markers, they were treated as missing and excluded from specific analyses. Pearson correlation coefficients were determined to assess correlations between markers of bacterial translocation.

The primary analyses consisted of multivariable conditional logistic regression models conditioning on the matching pair to estimate odds ratios (OR) and 95% confidence intervals (CI) between liver cancer risk and circulating bacterial translocation concentrations per doubling (using a log_2_ transformation) and quartiles of these markers based on control concentrations.^17^ In minimally adjusted models, we only conditioned models on matching factors. In multivariable adjusted models, we additionally adjusted for body mass index (BMI; <18.5, 18.5‐ < 25, 25‐ < 30, and ≥ 30 kg/m^2^), education level (some high school, high school graduate, some college, college degree, or postgraduate degree), smoking status (never, former, current), diabetes status (yes/no), estimated alcohol intake (non‐drinkers, 0.1–<10 g/day, 10–<20 g/day, 20–<40 g/day, and ≥40 g/day), and coffee consumption (non‐consumers, <1 cup/day, 1–2 cups/day, and ≥2 cups/day). Linear trend across the quartiles was assessed as a linear variable in the models where the quartiles were replaced with the median of the concentrations. To assess non‐linearity, a likelihood ratio test was used to compare models that treated bacterial translocation markers as continuous variables versus categorical quartile‐based models. Covariates with missing data were categorized into a separate missing category, with further details provided in the Supporting Information.

To evaluate heterogeneity in linear trends across studies, likelihood ratio tests were performed to compare *χ* ^2^ values from models with and without an interaction term between study and each bacterial translocation marker (modeled as a doubling in concentration). Heterogeneity in case‐defined factors (e.g., HCC vs. ICC, and time to liver cancer diagnosis: <10 years or ≥10 years) was assessed by fitting separate models for each subgroup, using a meta‐analytic approach in which controls were included only in analyses where their matched case was present. Subgroup analyses were conducted to examine differences in associations by liver cancer subtype (HCC vs. ICC) and by time from study recruitment to diagnosis (within 10 years vs. more than 10 years).

Heterogeneity in non‐case‐defined factors was assessed using likelihood ratio tests for *χ* ^2^ test of interaction between the bacterial translocation marker (modeled per doubling in concentration) and subgroup variables. Potential effect modification was examined by sex, BMI (~median; <27 kg/m^2^ vs. ≥27 kg/m^2^), alcohol intake (<20 g/day vs. ≥20 g/day), race (White vs. Black participants), diabetes status, and sample type provided (serum vs. plasma). Sensitivity analyses were conducted by excluding participants with evidence of HCV (positive HCV serology) or HBV infection (positive HBV antigen).

All statistical analyses were performed using Stata 18.0 or R 4.1.1. Two‐tailed p‐values were reported, with correction of multiple comparisons using the Benjamini–Hochberg procedure for false‐discovery rate (FDR) at a 0.05 level and 32 comparisons (based on the number of comparisons for doubling and quartile analyses).

## 4 RESULTS

Baseline characteristics of cases and controls are presented in Table 1 whereas a detailed description of baseline characteristics separated by study and case/control status can be found in Table S2. In comparison to controls, participants who developed liver cancer were more likely to have a higher BMI, live with diabetes, drink more alcohol, and be a current smoker.

**TABLE 1:** Baseline characteristics of liver cancer cases and selected controls.

	Cases (*n* = 867)	Controls (*n* = 867)
Sex^a^		
Males	364 (42.0%)	364 (42.0%)
Females	503 (58.0%)	503 (58.0%)
Age, years—Mean, SD^a^	60.0 (9.71)	59.9 (9.68)
Race/ethnicity^a^		
White	625 (72.1%)	625 (72.1%)
Black	153 (17.6%)	154 (17.8%)
Asian/Pacific Islander	33 (3.8%)	32 (3.7%)
American Indian/Alaska Native	4 (0.5%)	4 (0.5%)
Other	40 (4.6%)	42 (4.8%)
Body mass index, kg/m^2^—Mean, SD	28.1 (5.65)	27.5 (5.34)
Diabetes—Yes	133 (15.3%)	63 (7.3%)
Smoking		
Never	301 (34.7%)	357 (41.2%)
Former	345 (39.8%)	333 (38.4%)
Current	213 (24.6%)	165 (19.0%)
Alcohol intake, g/day—Mean, SD	18.5 (72.81)	11.7 (46.54)
Coffee intake, cups/day—Mean, SD	1.5 (1.73)	1.6 (1.75)
Education		
Some high school or less	114 (13.1%)	93 (10.7%)
High school degree	173 (20.0%)	190 (21.9%)
Some college or vocational training	255 (29.4%)	227 (26.2%)
College degree	137 (15.8%)	138 (15.9%)
Post‐college	162 (18.7%)	196 (22.6%)
Anti‐HCV positive^b^	54 (18.9%)	9 (3.4%)
HBsAg positive^b^	12 (4.2%)	1 (0.4%)
Time to diagnosis, years—Mean, SD	12.1 (7.5)	–

*Note*: Values are *N* (%) unless otherwise specified.

^a^ Variables were matched at study design.

^b^ A total of 552 participants (286 cases) had known anti‐HCV and HBsAg status.

For the liver cancer cases, blood collection preceded diagnosis by a mean of 12.1 years (SD = 7.5) across all cohorts. The mean duration between blood collection and diagnosis varied between cohorts with a range from 3.0 years in BWHS to 25.3 years in CLUE (Table S2).

Most of the bacterial translocation immunological markers were not strongly correlated with one another (*r* = −0.24 to 0.28), except for anti‐flagellin IgA and anti‐LPS IgA (*r* = 0.79; Figure S1). Concentrations of the bacterial translocation markers across each study and by case and control status are presented in Figure S2.

### 4.1 Associations of bacterial translocation immunological markers and liver cancer risk

**FIGURE 1:**
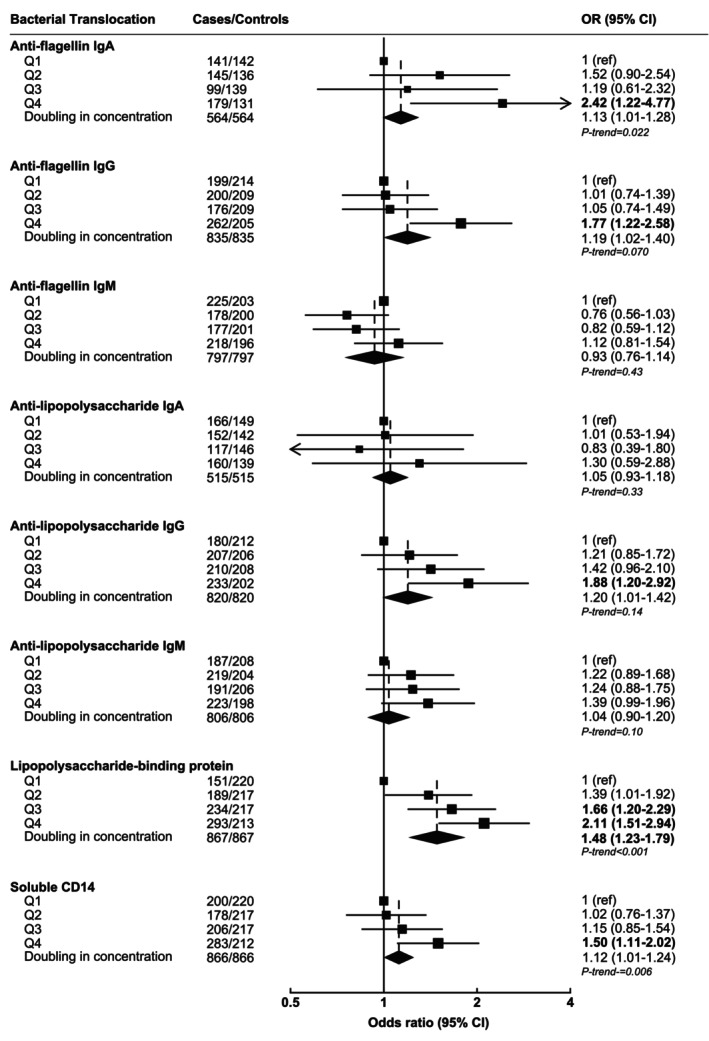
Odds ratios and 95% confidence intervals between circulating bacterial translocation concentrations and liver cancer risk by quartiles of concentrations and per doubling in concentrations. Multivariable conditional logistic regression models were conditioned on age, sex, date of blood draw, and cohort, and further adjusted for body mass index, education, smoking status, diabetes status, alcohol intake, and coffee intake. P‐trend was estimated by modeling the quartile variable as a linear variable and obtaining the *p*‐value from this estimate. Bolded odds ratio and 95% confidence intervals indicate bacterial translocation associations meeting correction for false discovery rate. Box size is proportional to the number of cases observed within each quartile. CI, confidence intervals; OR, odds ratio.

Associations, by quartiles and per doubling in concentrations, between concentrations of immunological markers of anti‐flagellin, anti‐LPS, LBP, and sCD14, and liver cancer risk are presented in Figure 1. Minimally adjusted associations between bacterial translocation immunological markers and liver cancer risk are presented in Figure S3.

Anti‐flagellin IgA and IgG concentrations were both associated with higher liver cancer risk (OR_per doubling_ for anti‐flagellin IgA = 1.13, 95% CI: 1.01–1.28 and anti‐flagellin IgG = 1.19, 1.02–1.40; Figure 1). Anti‐LPS IgG was also associated with higher liver cancer risk (OR_per doubling_ = 1.20, 1.01–1.42). LBP and sCD14 concentrations were also positively associated with liver cancer risk (OR_per doubling_ = 1.48, 1.23–1.79 and 1.12, 1.01–1.24, respectively). For doubling analyses, however, only LBP associations were statistically significant after correction for multiple comparisons. No statistically significant associations were observed between anti‐flagellin IgM, anti‐LPS IgA, and anti‐LPS IgM concentrations and risk of liver cancer.

In quartile analyses, associations were stronger comparing the highest vs. lowest quartile for anti‐flagellin IgA and IgG, anti‐LPS IgG, LBP, and sCD14 concentrations and risk of liver cancer (Figure 1). There was no evidence of non‐linearity for most of the bacterial translocation markers (all p‐value for non‐linearity >0.10), apart from anti‐flagellin IgG where there was some suggestion of non‐linearity (*p* = .04). Associations were generally consistent across studies (Figure S4) except for LBP and sCD14 where some heterogeneity was observed (*p* = .002 and .003, respectively).

### 4.2 Subtypes and sensitivity analyses

**FIGURE 2:**
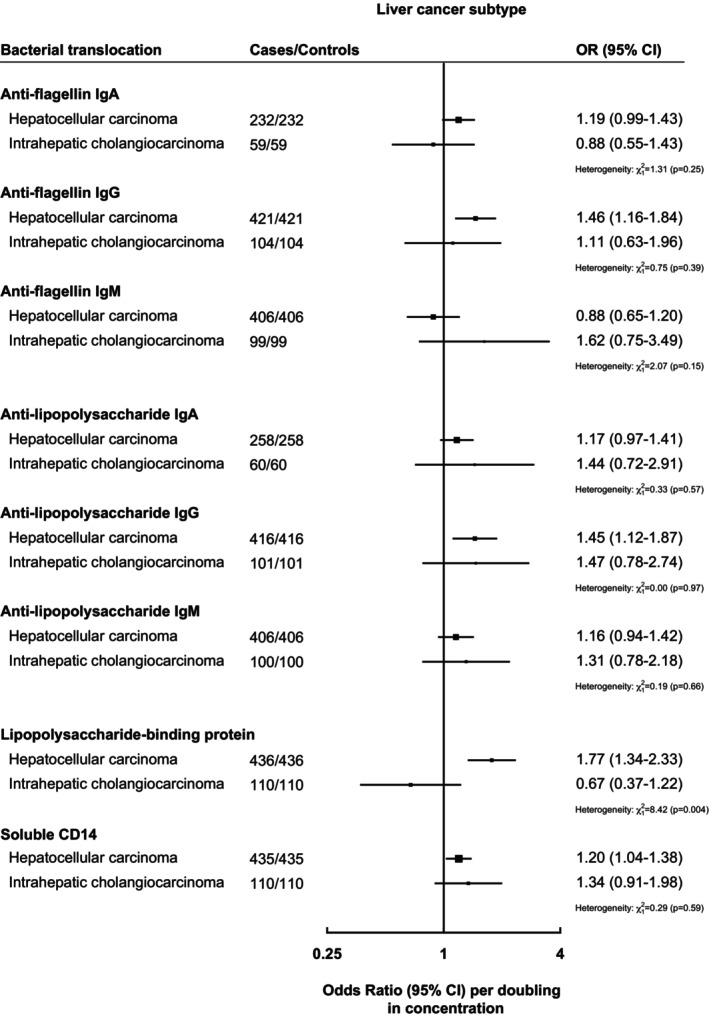
Odds ratios and 95% confidence intervals between circulating bacterial translocation concentrations and risk of subtypes of liver cancer: Hepatocellular carcinoma or intrahepatic cholangiocarcinoma. Multivariable conditional logistic regression models were conditioned on age, sex, time at blood draw, and cohort, and further adjusted for body mass index, education, smoking status, diabetes status, alcohol intake, and coffee intake. *χ* ^2^ and *p*‐values for heterogeneity were determined from Wald test for heterogeneity comparing odds ratio and standard errors between circulating bacterial translocation concentration (modeled per doubling in concentrations) and HCC versus ICC. Box size is proportional to the inverse variance within each subgroup.

There was no statistically significant evidence of heterogeneity for bacterial translocation markers and subtypes of liver cancer (HCC vs. ICC) except for LBP, which was positively associated with HCC (OR per doubling in concentration: 1.77, 1.34–2.33) but not with ICC (OR per doubling: 0.66, 0.37–1.22; *p*‐heterogeneity = .004; Figure 2).

**FIGURE 3:**
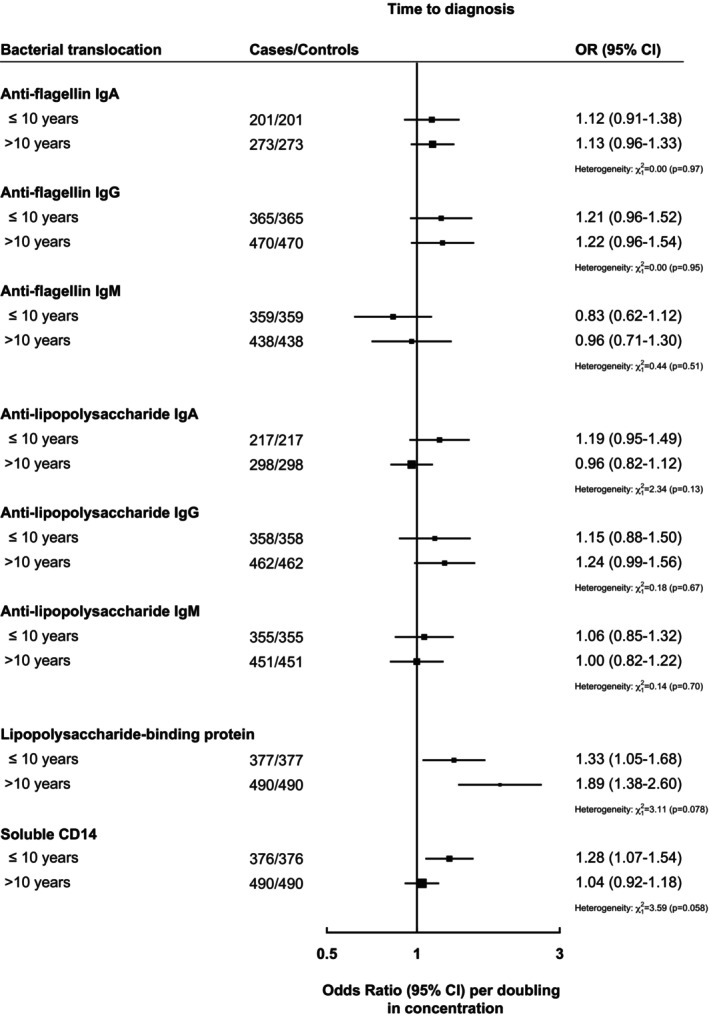
Odds ratios and 95% confidence intervals between circulating bacterial translocation concentrations and time between blood draw and liver cancer diagnosis. Multivariable conditional logistic regression models were conditioned on age, sex, time at blood draw, and cohort, and further adjusted for body mass index, education, smoking status, diabetes status, alcohol intake, and coffee intake. *χ* ^2^ and *p*‐values for heterogeneity were determined from Wald test for heterogeneity comparing odds ratio and standard errors between circulating bacterial translocation concentration (modeled per doubling in concentrations) and time to liver cancer diagnosis (≤10 years vs. >10 years). Box size is proportional to the inverse variance within each subgroup.

There was no statistically significant evidence of heterogeneity when we compared liver cancer cases diagnosed ≤10 years after blood collection with cases diagnosed >10 years after blood collection, although associations for LBP were stronger for liver cancer cases diagnosed >10 years after blood collection (Figure 3).

There was no evidence of heterogeneity between bacterial translocation markers and liver cancer risk by strata of sex, body mass index, alcohol intake, diabetes status, or sample type (Figures S5 and S6). We did, however, observe for sCD14 that there was some evidence of heterogeneity by race, in which associations were stronger for Black individuals (OR per doubling: 1.63, 1.22–2.16) than for White individuals (OR per doubling: 1.04, 0.93–1.17; *p*‐value for interaction = .002).

When participants who were positive for HBV or HCV were removed (*n* = 69 participants) along with their matched participant, associations generally remained the same, with some attenuation specifically for anti‐flagellin IgG and sCD14, although estimates did not significantly differ from the main multivariable models (Figure S7).

## 5 DISCUSSION

In this large prospective study including 12 United States cohorts, we found that higher circulating concentrations of several bacterial translocation markers were positively associated with liver cancer risk. Specifically, higher concentrations of LBP, anti‐LPS IgG, anti‐flagellin IgG, and sCD14 were all positively associated with the risk of liver cancer. Circulating LBP concentrations were positively associated with HCC, but not ICC, and associations for LBP were stronger for liver cancer cases diagnosed >10 years after blood collection. Other associations were largely consistent across subgroups.

Our findings add to the previous research that examined bacterial translocation markers and liver cancer risk.^17^, ^18^, ^21^ The three prior prospective studies had smaller sample sizes (<250 liver cancer cases) compared with the present study. Among the prior studies, only one, an analysis our group conducted in two Taiwanese cohorts, found that LBP was statistically significantly associated with HBV‐related liver cancer risk (OR per doubling in concentration = 1.93).^17^ In contrast, an analysis our group conducted among male Finnish participants who smoked found no significant association between LBP and liver cancer risk,^18^ and an analysis conducted in the Europe‐based EPIC cohort did not measure LBP concentrations.^21^ Consistent with our findings, the previous prospective studies observed significant positive associations with anti‐flagellin IgA, with estimates being approximately the same as our current analysis (OR_Q4 vs. Q1_ ~2–3)^17^, ^18^, ^21^; however, only the EPIC analysis of 139 cases and 139 controls found a positive association between anti‐LPS IgG and liver cancer risk.^21^

Our finding of an association between LBP concentrations and HCC, but not ICC, suggests potential specificity for the development of HCC. LBP is primarily synthesized by hepatocytes in response to bacterial infection and binds to LPS and then CD14 to initiate an immune response. Elevated LBP concentrations have been previously associated with metabolic dysfunction–associated steatotic liver disease (MASLD)^36^ and cirrhosis.^37^, ^38^, ^39^ Animal models have also suggested the importance of LBP in liver disease; for example, LBP knockout mice do not develop MASLD in comparison to wild‐type mice who are fed a high‐fat, fructose, and cholesterol diet.^40^ We also observed that associations for LBP and liver cancer risk were stronger for cases diagnosed >10 years after blood collection. This finding may suggest that LBP is an important aetiological factor involved in hepatocarcinogenesis rather than a marker of liver dysfunction. This is hard to determine, however, as underlying liver disease, which is associated with higher LBP concentrations,^36^, ^37^, ^38^ can begin many years before the diagnosis of HCC.^41^ In addition, some animal studies have suggested that LBP may exhibit protective properties against MASLD under non‐obesogenic or standard environmental conditions.^42^ Taken together, LBP, a known mediator between dietary intake, gut permeability, bacterial infection, and liver disorders, may be implicated in early molecular mechanisms of liver cancer development, in particular HCC.

We observed that anti‐LPS IgG and anti‐flagellin IgG and IgA were positively associated with liver cancer risk, with no evidence of heterogeneity between HCC versus ICC. In contrast, we did not observe any associations between anti‐LPS IgA and liver cancer risk. IgA is the predominant antibody at mucosal surfaces (such as the gut), where it plays a role in neutralizing pathogens locally, whereas IgG predominates systemically and provides long‐term protection against pathogens that have breached mucosal barriers. The positive associations observed with systemic LPS IgG but not IgA may suggest that bacterial translocation and systemic immune activation, rather than localized gut immune responses, are key mechanisms linking gut permeability to liver cancer development. We also did not observe any associations with IgM antibodies to anti‐flagellin or anti‐LPS with liver cancer risk. This finding is consistent with the known immunological response, as IgM antibodies are typically first produced during an acute infection to a novel pathogen.^43^ In contrast, gut barrier dysfunction may be a chronic condition involving prolonged exposure to gram‐negative bacteria.^9^ As a result, individuals with gut barrier impairment could experience sustained immune activation, leading to elevated levels of IgG antibodies.

Experimental animal models have suggested that chronic bacterial translocation may promote hepatocarcinogenesis through sustained inflammation. LPS accumulation has been shown to activate inflammation and hepatocarcinogenesis via toll‐like receptor 4 (TLR4).^16^, ^44^, ^45^ Similarly, in murine models, high‐dose flagellin exposure has been shown to activate toll‐like receptor 5 (TLR5) signaling, promoting oxidative stress and inflammation, leading to liver injury.^15^ In addition, animal models have shown that antibiotics reduce liver fibrosis severity^46^ and HCC tumor formation,^16^, ^47^ further suggesting a role of gut‐derived bacterial products in hepatocarcinogenesis. Dysbiosis of gut microbiota has also been suggested to influence the risk of ICC,^48^, ^49^ potentially through TLR4‐dependent mechanisms to promote the formation of ICC.^49^, ^50^

Lifestyle factors such as alcohol intake and certain dietary patterns have been associated with increased gut permeability and potential bacterial translocation.^51^, ^52^ These same exposures are risk factors associated with a higher risk of liver cancer,^53^, ^54^, ^55^ suggesting that systemic bacterial translocation may be a critical biological mediator linking lifestyle behaviors to liver carcinogenesis. Additional research is needed, however, to explore these mechanisms.

There were many strengths to the current study. To our knowledge, this study is the largest to explore the associations between bacterial translocation markers and liver cancer risk. As a result, we were able to examine liver cancer subtypes, HCC versus ICC, to evaluate whether associations differed. The bacterial translocation markers were also measured at the same time in the same laboratories with the case and control pairs run in the same batches; therefore, minimizing any inter‐lab differences in measurement. The markers were examined in pre‐diagnostic samples, which on average were collected 12 years before liver cancer diagnosis, minimizing the potential of reverse causality, and we observed no statistically significant difference between analyses by time to diagnosis. We were also able to adjust for important confounders, as each study ascertained detailed sociodemographic information from participants.

While the study had numerous strengths, there were also several limitations. The immunological markers were measured at a single time point, and the marker concentrations may change over time with exposure to bacteria from the gut, which may introduce non‐differential measurement error and attenuate the true associations. Stability of the immunoglobulins is unclear as these can wax and wane over time; however, studies looking at LBP and sCD14 concentrations over time suggested moderate stability over 6–9 months (intraclass coefficient = 0.52 and 0.60, respectively).^56^, ^57^ Collection and storage of the samples also differed between the various studies, and some studies stored sera while others stored plasma. While differences in sample type may result in differences in concentrations, this was taken into consideration in the design phase as all the case and control pairs were the same sample type. Also, due to the observational nature of the study, residual and unmeasured confounding may be present, which may influence the findings, particularly for covariates that were not available or measured such as dietary intake and antibiotic use.

## 6 CONCLUSION

These findings support a role in gut barrier dysfunction in hepatocarcinogenesis. Associations were generally consistent by liver cancer subtypes, HCC or ICC, except for LBP, which was positively associated with HCC and not associated with ICC. LBP concentrations appeared to be more strongly associated with liver cancer diagnosed >10 years after blood collection, although there was no significant evidence of heterogeneity. Further research into the underlying causes and risk factors of bacterial translocation, as well as the development of effective interventions, may identify promising strategies for reducing liver cancer risk.

## AUTHOR CONTRIBUTIONS

**Cody Z. Watling:** Investigation; writing – original draft; methodology; validation; visualization; formal analysis. **Peter T. Campbell:** Investigation; writing – review and editing. **Barry I. Graubard:** Investigation; writing – review and editing. **Yanyu Wang:** Investigation; writing – review and editing. **Andrew T. Gewirtz:** Investigation; writing – review and editing. **Xuehong Zhang:** Investigation; writing – review and editing. **Matthew J. Barnett:** Investigation; writing – review and editing. **Julie E. Buring:** Investigation; writing – review and editing. **Yu Chen:** Investigation; writing – review and editing. **A. Heather Eliassen:** Investigation; writing – review and editing. **J. Michael Gaziano:** Investigation; writing – review and editing. **Jonathan N. Hofmann:** Investigation; writing – review and editing. **Wen‐Yi Huang:** Investigation; writing – review and editing. **Jae H. Kang:** Investigation; writing – review and editing. **Jill Koshiol:** Investigation; writing – review and editing. **Erikka Loftfield:** Investigation; writing – review and editing. **I‐Min Lee:** Investigation; writing – review and editing. **Steven C. Moore:** Investigation; writing – review and editing. **Lorelei A. Mucci:** Investigation; writing – review and editing. **Marian L. Neuhouser:** Investigation; writing – review and editing. **Christina C. Newton:** Investigation; writing – review and editing. **Mark P. Purdue:** Investigation; writing – review and editing. **Howard D. Sesso:** Investigation; writing – review and editing. **Martha Shrubsole:** Investigation; writing – review and editing. **Rashmi Sinha:** Investigation; writing – review and editing. **Lesley Tinker:** Investigation; writing – review and editing. **Matthew Triplette:** Investigation; writing – review and editing. **Caroline Y. Um:** Investigation; writing – review and editing. **Kala Visvanathan:** Investigation; writing – review and editing. **Eleanor L. Watts:** Investigation; writing – review and editing. **Jean Wactawski‐Wende:** Investigation; writing – review and editing. **Walter Willett:** Investigation; writing – review and editing. **Fen Wu:** Investigation; writing – review and editing. **Wei Zheng:** Investigation; writing – review and editing. **Dinesh Barupal:** Investigation; writing – review and editing. **Jessica L. Petrick:** Investigation; writing – review and editing. **Katherine A. McGlynn:** Investigation; supervision; resources; project administration; writing – review and editing; funding acquisition; methodology; conceptualization; data curation.

## CONFLICT OF INTEREST STATEMENT

Lorelei A. Mucci holds equity interest in Convergent Therapeutics and received research funding (to Harvard University) from Astra Zeneca. These were unrelated to the present study. All other authors have no conflicts of interest to report.

## ETHICS STATEMENT

Each participating cohort obtained ethical approval from its respective institutional review board, and all participants provided informed consent at study enrollment.

## Supporting information


**Table S1.** Characteristics of cohort recruitment, number of liver cancer cases, and blood collection across included cohorts.
**Table S2**. Baseline characteristics of participants across cohorts between cases and controls.
**Figure S1**. Correlation matrix between immunological bacterial translocation markers.
**Figure S2**. Concentrations of bacterial translocation markers across the included studies and by case and control status.
**Figure S3**. Minimally‐adjusted and multivariable adjusted odds ratios and 95% confidence intervals for bacterial.
**Figure S4**. Study‐specific odds ratios and 95% confidence intervals for bacterial translocation concentrations per.
**Figure S5**. Multivariable‐adjusted odds ratios and 95% confidence intervals for circulating bacterial translocation.
**Figure S6**. Multivariable‐adjusted odds ratios and 95% confidence intervals for circulating bacterial translocation.
**Figure S7**. Multivariable‐adjusted odds ratios and 95% confidence intervals per doubling in concentrations of bacterial.

## Data Availability

The data that support the findings of this study are available from the corresponding author upon reasonable request.
