# Supplementary material for: Pre‐diagnostic immunological markers of bacterial translocation and liver cancer risk: A nested case–control analysis of 12 prospective cohorts
Source: Int J Cancer. 2025 Oct 23;158(7):1801–12. doi: 10.1002/ijc.70201 (PMC12875164; doi:10.1002/ijc.70201)
Supplement: Supplementary file 1 — Table S1. Characteristics of cohort recruitment, number of liver cancer cases, and blood collection across included cohorts. Table S2. Baseline characteristics of participants across cohorts between cases and controls. Figure S1. Correlation matrix between immunological bacterial translocation markers. Figure S2. Concentrations of bacterial translocation markers across the included studies and by case and control status. Figure S3. Minimally‐adjusted and multivariable adjusted odds ratios and 95% confidence intervals for bacterial. Figure S4. Study‐specific odds ratios and 95% confidence intervals for bacterial translocation concentrations per. Figure S5. Multivariable‐adjusted odds ratios and 95% confidence intervals for circulating bacterial translocation. Figure S6. Multivariable‐adjusted odds ratios and 95% confidence intervals for circulating bacterial translocation. Figure S7. Multivariable‐adjusted odds ratios and 95% confidence intervals per doubling in concentrations of bacterial. [file IJC-158-1801-s001.docx]

**Pre-diagnostic immunological markers of bacterial translocation and liver cancer risk: a nested case-control analysis of 12 prospective cohorts**

Cody Z Watling, Peter T Campbell, Barry I Graubard, Yanyu Wang, Andrew T Gewirtz, Xuehong Zhang, Matthew J Barnett, Julie E Buring, Yu Chen, A. Heather Eliassen, J Michael Gaziano, Jonathan N Hofmann, Wen-Yi Huang, Jae H Kang, Jill Koshiol, Erikka Loftfield, I-Min Lee, Steven C Moore, Lorelei A Mucci, Marian L Neuhouser, Christina C Newton, Mark P Purdue, Howard D Sesso, Martha Shrubsole, Rashmi Sinha, Lesley Tinker, Matthew Triplette, Caroline Y Um, Kala Visvanathan, Eleanor L Watts, Jean Wactawski-Wende, Walter Willett, Fen Wu, Wei Zheng, Dinesh Barupal, Jessica L Petrick, Katherine A McGlynn

Table of Contents

[Supplementary Materials and Methods 3](#_Toc211618550)

[Antibodies to LPS and flagellin 3](#_Toc211618551)

[Measurements of lipopolysaccharide-binding protein (LBP) and soluble CD14 (sCD14) 3](#_Toc211618552)

[Covariate classification 4](#_Toc211618553)

[Supplementary Tables 6](#_Toc211618554)

[Table S1. Characteristics of cohort recruitment, number of liver cancer cases, and blood collection across included cohorts. 6](#_Toc211618555)

[Table S2. Baseline characteristics of participants across cohorts between cases and controls 7](#_Toc211618556)

[Supplementary Figures 9](#_Toc211618557)

[Figure S1. Correlation matrix between immunological bacterial translocation markers 9](#_Toc211618558)

[Figure S2. Concentrations of bacterial translocation markers across the included studies and by case and control status 10](#_Toc211618559)

[Figure S3. Minimally-adjusted and multivariable adjusted odds ratios and 95% confidence intervals for bacterial translocation concentrations and risk of liver cancer by quartiles and per doubling in concentrations. 11](#_Toc211618560)

[Figure S4. Study-specific odds ratios and 95% confidence intervals for bacterial translocation concentrations per doubling in concentrations and risk of liver cancer. 12](#_Toc211618561)

[Figure S5. Multivariable-adjusted odds ratios and 95% confidence intervals for circulating bacterial translocation concentrations and risk of liver cancer per doubling in concentrations by subgroups of interest 13](#_Toc211618562)

[Figure S6. Multivariable-adjusted odds ratios and 95% confidence intervals for circulating bacterial translocation concentrations and risk of liver cancer per doubling in concentrations by subgroups of interest 14](#_Toc211618563)

[Figure S7. Multivariable-adjusted odds ratios and 95% confidence intervals per doubling in concentrations of bacterial translocation markers and risk of liver cancer removing individuals who had positive hepatitis C or B virus serology. 15](#_Toc211618564)

[References 16](#_Toc211618565)

# Supplementary Materials and Methods

## Antibodies to LPS and flagellin

ELISA plates (Costar™ 3590) were coated overnight with laboratory-produced flagellin or purified Escherichia coli LPS. Serum samples, diluted at 1:200, were then added to the coated wells. Following incubation and washing, wells were treated with horseradish peroxidase-conjugated anti-IgM, anti-IgA, or anti-IgG. Total immunoglobulin levels were quantified using the colorimetric peroxidase substrate tetramethylbenzidine, with optical density measured at 450 nm and 540 nm. Data are reported as optical density values correcting by subtracting background. For quality control, two duplicate samples were measured in each batch. The between-batch coefficient of variation (CV) was between 16.1-40.8% whereas the within batch CV was between 9.0-28.4%. The intraclass correlation coefficient was between 0.82-0.99 for all measured antibodies.

## Measurements of lipopolysaccharide-binding protein (LBP) and soluble CD14 (sCD14)

LBP was quantified using the R&D Systems DuoSet ELISA kit (Cat# DY870–05 and DY008). 96-well microplates were coated overnight with a human LBP capture antibody, followed by the application of serum or plasma samples diluted 1:1000. After incubation and washing, wells were treated with streptavidin conjugated to horseradish peroxidase. sCD14 levels were measured using the R&D Systems Quantikine kit (Cat# CD140). This assay utilizes a 96-well polystyrene microplate pre-coated with a monoclonal antibody specific to human CD14. Samples, diluted 1:1000 in the manufacturer’s recommended diluent, were applied to the coated wells. Following incubation and washing, wells were incubated with a polyclonal antibody specific to human CD14, conjugated to horseradish peroxidase with preservatives. For both LBP and sCD14, optical densities were measured at 450 nm and 540 nm. All samples were tested in duplicate, and the average value was used for analysis. Final concentrations were determined using a standard curve and reported in μg/mL for both LBP and sCD14. Two quality control samples were measured in each batch. The between-batch CV was 11.7% for SCD14 and 18.5% for LBP, within batch CV was 8.1% for SCD14 and 6.1% for LBP, whereas the intraclass correlation coefficient was 0.88 for SCD14 and 0.91 for LBP.

## Covariate classification

*Body mass index*

Each cohort provided information on body mass index (BMI) from reported weight and height. From this we determined the BMI of each participant and categorized them into the following groups: <18.5, 18.5-<25, 25-<30 and 30+ kg/m^2^. If a participant was missing, they were categorized into a missing category (6.3% of all participants).

*Education*

Each cohort provided information on educational attainment and from this we assigned participants into the following categories: some high school, high school degree, some college, college degree, or post college degree. Participants with missing information on education were assigned into a missing category (2.8% of all participants).

*Smoking status*

Each cohort provided information on smoking status of participants, and we assigned them into the following categories: never, former, current. Participants with missing information on smoking were assigned into a missing category (1.2% of all participants).

*Diabetes status*

Each cohort provided information on diabetes status at recruitment of participants and from this we assigned them as living with diabetes (yes) or not living with diabetes (no). Participants with missing information on diabetes status were assigned into a missing category (6.2% of all participants).

*Alcohol intake*

Each cohort provided information estimated alcohol intake (g/day of ethanol) at recruitment of participants and from this we assigned them into the following categories: non-drinkers, 0.1-<10 g/day, 10-<20 g/day, 20-<40 g/day and 40+ g/day. Participants with missing information on alcohol intake were assigned into a missing category (10.5% of all participants).

*Coffee intake*

Each cohort provided information estimated coffee intake (cups/day) at recruitment of participants and from this we assigned them into the following categories: non-consumers, <1 cup/day, 1-2 cups/day and 2+ cups/day. Participants with missing information on coffee intake were assigned into a missing category (13.1% of all participants).

# Supplementary Tables

| Table S1. Characteristics of cohort recruitment, number of liver cancer cases, and blood collection across included cohorts. | | | | | | | | | | | |
| --- | --- | --- | --- | --- | --- | --- | --- | --- | --- | --- | --- |
| **Cohort** | **Year(s) of Enrollment** | **Liver Cancer Cases** | **Liver Cancer - Male** | **Liver Cancer - Female** | **HCC** | **ICC** | **Blood Collection Years** | **Sample Type** | **Fasting Status** | **Storage Temp** | **Time from Draw to Frozen** |
| BWHS | 1995 | 5 | - | 5 | 2 | 0 | 2013-2017 | plasma | Non-fasting | -80°C | Frozen ≤28 hours |
| CARET | 1985-1994 | 53 | 48 | 5 | 41 | 6 | 1985-1990 | serum | Non-fasting | -70°C | Frozen within 2 hours |
| CLUE | CLUE I: 1974 CLUE II: 1989 | 43 | 28 | 15 | 28 | 12 | CLUE I: 1974 CLUE II: 1989 | serum | Non-fasting | -70°C | <24 hours (usually 1-2 h) |
| CPS-II | 1992-1993 | 48 | 31 | 17 | 27 | 9 | 1998-2001 | serum | Non-fasting | -130°C | Overnight mailing |
| HPFS | 1986 | 20 | 20 | - | 5 | 4 | 1993-1995 | plasma | 75% fasting | -130°C | 97% arrived ≤ 26 hrs |
| NHS | 1976 | 33 | - | 33 | 12 | 4 | 1989-1990 | plasma | 75% fasting | -130°C | 97% arrived ≤ 26 hrs |
| NYUWHS | 1985-1991 | 27 | - | 27 | 5 | 5 | 1985-1991 | serum | Non-fasting | -80°C | Frozen immediately |
| PHS | PHS I: 1981 PHS II: 1997 | 16 | 16 | - | 6 | 0 | PHS I: 1982-1983 PHS II: 1997 | plasma | 44% fasted 4+ hours, 26% fasted 8+ hours | -80°C | Overnight mailing |
| PLCO | 1993-2001 | 172 | 130 | 42 | 110 | 30 | 1993-2001 | serum | Non-fasting | -70°C or -157°C | Frozen within 2-4 hours |
| SCCS | 2002-2009 | 127 | 91 | 36 | 98 | 11 | 2002-2004 | serum | Non-fasting | -86°C | Frozen <24 hours |
| WHI | 1993-1998 | 296 | - | 296 | 100 | 29 | 1993 | serum | 12+ hours fast | -80°C | Frozen within  2 hrs |
| WHS | 1992-1995 | 27 | - | 27 | 2 | 0 | 1992 | plasma | Overnight fast | -170°C | Frozen 30-36 hrs |
| Abbreviations: BWHS, Black Women’s Health Study; CARET, Beta-Carotene and Retinol Efficacy Trial; CLUE, Campaign against Cancer and Stroke Study; CPS-II, Cancer Prevention Study-II Nutrition Cohort; HCC, hepatocellular carcinoma; HPFS, Health Professionals Follow-Up Study; ICC, intrahepatic cholangiocarcinoma; NHS, Nurses’ Health Study; NYU New York University Women’s Health Study; PHS, Physicians’ Health Study; PLCO, Prostate, Lung, Colorectal and Ovarian Cancer Screening Trial; SCCS, Southern Community Cohort Study; WHI, Women’s Health Initiative; WHS, Women’s Health Study. | | | | | | | | | | | |

| Table S2. Baseline characteristics of participants across cohorts between cases and controls | | | | | | | | | | | | | | | |
| --- | --- | --- | --- | --- | --- | --- | --- | --- | --- | --- | --- | --- | --- | --- | --- |
|  |  |  | **Sex** | |  |  |  |  |  |  |  |  |  |  |  |
|  |  | **N** | **Female** | **Males** | **Age at recruitment** | **Ethnicity:**  **White** | **BMI kg/m^2^** | **Living with diabetes** | **Current Smoker** | **Alcohol intake >40 g/day** | **Coffee intake >2 cups/day** | **College Degree** | **Anti-HCV serology positive** | **HBsAg serology positive** | **Time to diagnosis, years** |
| BHWS | Case | 5 | 5 (100%) | - | 66.8 (12.4) | 0 (0%) | 33.5 (2.9) | 1 (20%) | 0 (0%) | - | - | 2 (40%) | - | - | 3.0 (1.2) |
|  | Control | 5 | 5 (100%) | - | 66.8 (12.4) | 0 (0%) | 31.2 (7.2) | 0 (0%) | 1 (20%) | - | - | 1 (20%) | - | - | - |
| CARET | Case | 53 | 5 (9%) | 48 (91%) | 58.6 (6.3) | 45 (85%) | 28.9 (4.8) | 8 (15%) | 29 (55%) | 7 (13%) | 37 (70%) | 6 (11%) | - | - | 13.1 (5.8) |
|  | Control | 53 | 5 (9%) | 48 (91%) | 58.7 (6.2) | 45 (85%) | 27.8 (5.1) | 3 (6%) | 31 (58%) | 3 (6%) | 33 (62%) | 8 (15%) | - | - | - |
| CLUE | Case | 43 | 15 (35%) | 28 (65%) | 46.2 (16.8) | 43 (100%) | - | - | 14 (33%) | - | - | - | - | - | 25.3 (11.6) |
|  | Control | 43 | 15 (35%) | 28 (65%) | 46.2 (16.6) | 43 (100%) | - | - | 14 (33%) | - | - | - | - | - | - |
| CPS-II | Case | 48 | 17 (35%) | 31 (65%) | 61.8 (5.5) | 47 (98%) | 28.1 (5.7) | 3 (6%) | 6 (12%) | 5 (10%) | 17 (35%) | 17 (35%) | 2 (4%) | 0 (0%) | 8.6 (4.2) |
|  | Control | 48 | 17 (35%) | 31 (65%) | 61.8 (5.6) | 47 (98%) | 27.0 (4.1) | 5 (10%) | 5 (10%) | 2 (4%) | 19 (40%) | 14 (29%) | 0 (0%) | 0 (0%) | - |
| HPFS | Case | 20 | - | 20 (100%) | 56.4 (9.2) | 19 (95%) | 27.5 (3.7) | 1 (5%) | 2 (10%) | 3 (15%) | 7 (35%) | 20 (100%) | 0 (0%) | 0 (0%) | 18.6 (4.8) |
|  | Control | 20 | - | 20 (100%) | 56.1 (8.9) | 19 (95%) | 24.7 (2.7) | 1 (5%) | 0 (0%) | 1 (5%) | 5 (25%) | 20 (100%) | 0 (0%) | 0 (0%) | - |
| NHS | Case | 33 | 33 (100%) | - | 49.4 (7.7) | 30 (91%) | 25.4 (3.6) | 2 (6%) | 4 (12%) | 0 (0%) | 15 (45%) | 24 (73%) | 4 (12%) | 0 (0%) | 22.9 (7.2) |
|  | Control | 33 | 33 (100%) | - | 49.6 (7.2) | 30 (91%) | 23.6 (3.4) | 0 (0%) | 5 (15%) | 0 (0%) | 19 (58%) | 27 (82%) | 0 (0%) | 0 (0%) | - |
| NYUWHS | Case | 27 | 27 (100%) | - | 52.8 (7.4) | 12 (44%) | 24.9 (3.0) | 0 (0%) | 6 (22%) | 0 (0%) | - | 2 (7%) | 5 (19%) | 1 (4%) | 18.8 (7.5) |
|  | Control | 27 | 27 (100%) | - | 52.7 (7.5) | 12 (44%) | 26.4 (5.4) | 2 (7%) | 3 (11%) | 0 (0%) | - | 5 (19%) | 3 (11%) | 0 (0%) | - |
| PHS | Case | 16 | - | 16 (100%) | 57.4 (10.2) | 14 (88%) | 24.9 (3.6) | 0 (0%) | 1 (6%) | 0 (0%) | 4 (25%) | 16 (100%) | 3 (19%) | 4 (25%) | 16.1 (7.5) |
|  | Control | 16 | - | 16 (100%) | 57.3 (10.1) | 15 (94%) | 24.6 (1.6) | 0 (0%) | 0 (0%) | 0 (0%) | 4 (25%) | 16 (100%) | 0 (0%) | 0 (0%) | - |
| PLCO | Case | 172 | 42 (24.4%) | 130 (75.6%) | 63.1 (5.2) | 138 (80.2%) | 28.9 (5.1) | 39 (22.7%) | 33 (19.2%) | 17 (9.9%) | 61 (35.5%) | 28 (16.3%) | 19 (11.0%) | 3 (1.7%) | 9.4 (4.8) |
|  | Control | 172 | 42 (24.4%) | 130 (75.6%) | 63.0 (5.1) | 138 (80.2%) | 27.3 (4.6) | 13 (7.6%) | 18 (10.5%) | 17 (9.9%) | 70 (40.7%) | 29 (16.9%) | 4 (2.3%) | 1 (0.6%) | - |
| SCCS | Case | 127 | 36 (28.3%) | 91 (71.7%) | 53.0 (8.3) | 19 (15.0%) | 27.3 (6.0) | 35 (27.6%) | 87 (68.5%) | 35 (27.6%) | 7 (5.5%) | 5 (3.9%) | - | - | 7.3 (3.8) |
|  | Control | 127 | 36 (28.3%) | 91 (71.7%) | 53.0 (8.3) | 19 (15.0%) | 28.8 (6.1) | 25 (19.7%) | 65 (51.2%) | 18 (14.2%) | 11 (8.7%) | 9 (7.1%) | - | - | - |
| WHI | Case | 296 | 296 (100.0%) | - | 65.4 (6.8) | 232 (78.4%) | 28.9 (6.2) | 43 (14.5%) | 28 (9.5%) | 2 (0.7%) | 59 (19.9%) | 43 (14.5%) | 21 (7.1%) | 4 (1.4%) | 11.5 (5.8) |
|  | Control | 296 | 296 (100.0%) | - | 65.4 (6.8) | 232 (78.4%) | 28.2 (5.7) | 14 (4.7%) | 21 (7.1%) | 5 (1.7%) | 58 (19.6%) | 33 (11.1%) | 2 (0.7%) | 0 (0.0%) | - |
| WHS | Case | 27 | 27 (100%) | - | 56.0 (8.3) | 26 (96%) | 26.2 (4.9) | 1 (4%) | 3 (11%) | 0 (0%) | 13 (48%) | 6 (22%) | - | - | 15.3 (5.1) |
|  | Control | 27 | 27 (100%) | - | 55.8 (8.1) | 25 (93%) | 25.3 (3.9) | 0 (0%) | 2 (7%) | 0 (0%) | 18 (67%) | 3 (11%) | - | - | - |
| Values are mean (SD) or N (%).  Abbreviations: BMI, body mass index; BWHS, Black Women’s Health Study; CARET, Beta-Carotene and Retinol Efficacy Trial; CLUE, Campaign against Cancer and Stroke Study; CPS-II, Cancer Prevention Study-II Nutrition Cohort; HBV, hepatitis B virus; HCV, hepatitis C virus; HPFS, Health Professionals Follow-Up Study; N, number of participants; NHS, Nurses’ Health Study; NYU New York University Women’s Health Study; PHS, Physicians’ Health Study; PLCO, Prostate, Lung, Colorectal and Ovarian Cancer Screening Trial; SCCS, Southern Community Cohort Study; WHI, Women’s Health Initiative; WHS, Women’s Health Study. | | | | | | | | | | | | | | | |

# Supplementary Figures

## Figure S1. Correlation matrix between immunological bacterial translocation markers

Values represent Pearson correlation coefficients.

All concentrations were log transformed.

Figure S2. Concentrations of bacterial translocation markers across the included studies and by case and control status**.**

P-values are from one way analysis of variance for difference in concentrations between studies.

Abbreviations: BWHS, Black Women’s Health Study; CARET, Beta-Carotene and Retinol Efficacy Trial; CLUE, Campaign against Cancer and Stroke Study; CPS-II, Cancer Prevention Study-II Nutrition Cohort; HPFS, Health Professionals Follow-Up Study; LBP, Lipopolysaccharide-binding protein; NHS, Nurses’ Health Study; NYU New York University Women’s Health Study; PHS, Physicians’ Health Study; PLCO, Prostate, Lung, Colorectal and Ovarian Cancer Screening Trial; SCD14, soluble CD14; SCCS, Southern Community Cohort Study; WHI, Women’s Health Initiative; WHS, Women’s Health Study.

For antibodies to flagellin and lipopolysaccharide, these are optimal densities. For lipopolysaccharide-binding protein and soluble CD14 the concentration is in μg/mL.

## Figure S3. Minimally-adjusted and multivariable adjusted odds ratios and 95% confidence intervals for bacterial translocation concentrations and risk of liver cancer by quartiles and per doubling in concentrations.

Minimally adjusted models are only conditioned on matching variables (age, sex, ethnicity, cohort, time of blood draw).

Multivariable adjusted conditional logistic regression models were conditioned on age, sex, time at blood draw, and cohort, and further adjusted for body mass index, education, smoking status, diabetes status, alcohol intake, and coffee intake.

Concentration in controls represents the mean biomarker concentrations among control participants in each quartile. For antibodies to flagellin and lipopolysaccharide, these are optimal densities. For lipopolysaccharide-binding protein and soluble CD14 the concentrations are μg/mL.

## Figure S4. Study-specific odds ratios and 95% confidence intervals for bacterial translocation concentrations per doubling in concentrations and risk of liver cancer.

Multivariable conditional logistic regression models conditioned on the matching factors age, sex, time at blood draw, and cohort and further adjusted for body mass index, education, smoking status, diabetes status, alcohol intake, and coffee intake.

P-heterogeneity represents the p-value obtained by fitting a model with an interaction term between the bacterial translocation marker of interest (per doubling in concentration) and study variable (df=11).

## Figure S5. Multivariable-adjusted odds ratios and 95% confidence intervals for circulating bacterial translocation concentrations and risk of liver cancer per doubling in concentrations by subgroups of interest

Multivariable conditional logistic regression models conditioned on the matching factors age, sex, time at blood draw, and cohort further adjusted for body mass index (except when it was the subgroup of interest), education, smoking status, diabetes status, alcohol intake (except when it was subgroup of interest), and coffee intake.

P-value for heterogeneity obtained from likelihood ratio test comparing models with bacterial translocation variable (modelled per doubling in concertation) and interaction term between subgroup of interest to a model without an interaction term.

## Figure S6. Multivariable-adjusted odds ratios and 95% confidence intervals for circulating bacterial translocation concentrations and risk of liver cancer per doubling in concentrations by subgroups of interest

Multivariable conditional logistic regression models conditioned on the matching factors age, sex, time at blood draw, and cohort further adjusted for body mass index, education, smoking status, diabetes status (except when it was the subgroup of interest), alcohol intake and coffee intake.

P-value for heterogeneity obtained from likelihood ratio test comparing models with bacterial translocation variable (modelled per doubling in concertation) and interaction term between subgroup of interest to a model without an interaction term.

## Figure S7. Multivariable-adjusted odds ratios and 95% confidence intervals per doubling in concentrations of bacterial translocation markers and risk of liver cancer removing individuals who had positive hepatitis C or B virus serology.

Multivariable conditional logistic regression models conditioned on the matching factors age, sex, time at blood draw, and cohort further adjusted for body mass index, education, smoking status, diabetes status, alcohol intake, and coffee intake.

P-difference was determined from Wald test for heterogeneity comparing odds ratio and standard errors between circulating bacterial translocation marker of interest (modelled per doubling in concentrations) in multivariable adjusted models with HCV and HBV positive participants and models removing HCV and HBV positive participants.

# References

1 Rosenberg, L., Adams-Campbell, L. & Palmer, J. R. The Black Women's Health Study: a follow-up study for causes and preventions of illness. *J Am Med Womens Assoc (1972)* **50**, 56-58 (1995).

2 Omenn, G. S., Goodman, G., Thornquist, M., Grizzle, J., Rosenstock, L., Barnhart, S. *et al.* The beta-carotene and retinol efficacy trial (CARET) for chemoprevention of lung cancer in high risk populations: smokers and asbestos-exposed workers. *Cancer Res* **54**, 2038s-2043s (1994).

3 Cancer Epidemiology Descriptive Cohort Database. *Clue Cohort Study - Clue I (CLUE I)*, <https://cedcd.nci.nih.gov/cohort?id=134> (2023).

4 Calle, E. E., Rodriguez, C., Jacobs, E. J., Almon, M. L., Chao, A., McCullough, M. L. *et al.* The American Cancer Society Cancer Prevention Study II Nutrition Cohort: rationale, study design, and baseline characteristics. *Cancer* **94**, 500-511 (2002).

5 Grobbee, D. E., Rimm, E. B., Giovannucci, E., Colditz, G., Stampfer, M. & Willett, W. Coffee, caffeine, and cardiovascular disease in men. *N Engl J Med* **323**, 1026-1032 (1990).

6 Colditz, G. A., Manson, J. E. & Hankinson, S. E. The Nurses' Health Study: 20-year contribution to the understanding of health among women. *J Womens Health* **6**, 49-62 (1997).

7 Toniolo, P. G., Pasternack, B. S., Shore, R. E., Sonnenschein, E., Koenig, K. L., Rosenberg, C. *et al.* Endogenous hormones and breast cancer: a prospective cohort study. *Breast Cancer Res Treat* **18 Suppl 1**, S23-26 (1991).

8 Final report on the aspirin component of the ongoing Physicians' Health Study. *N Engl J Med* **321**, 129-135 (1989).

9 Gohagan, J. K., Prorok, P. C., Hayes, R. B. & Kramer, B. S. The Prostate, Lung, Colorectal and Ovarian (PLCO) Cancer Screening Trial of the National Cancer Institute: history, organization, and status. *Control Clin Trials* **21**, 251s-272s (2000).

10 Signorello, L. B., Hargreaves, M. K. & Blot, W. J. The Southern Community Cohort Study: investigating health disparities. *J Health Care Poor Underserved* **21**, 26-37 (2010).

11 Anderson, G. L., Manson, J., Wallace, R., Lund, B., Hall, D., Davis, S. *et al.* Implementation of the Women's Health Initiative study design. *Ann Epidemiol* **13**, S5-17 (2003).

12 Rexrode, K. M., Lee, I. M., Cook, N. R., Hennekens, C. H. & Buring, J. E. Baseline characteristics of participants in the Women's Health Study. *J Womens Health Gend Based Med* **9**, 19-27 (2000).
